# Safety, Immunogenicity and Antibody Persistence of Rift Valley Fever Virus Clone 13 Vaccine in Sheep, Goats and Cattle in Tanzania

**DOI:** 10.3389/fvets.2021.779858

**Published:** 2021-12-17

**Authors:** Calvin Sindato, Esron D. Karimuribo, Emmanuel S. Swai, Leonard E. G. Mboera, Mark M. Rweyemamu, Janusz T. Paweska, Jeremy Salt

**Affiliations:** ^1^National Institute for Medical Research, Tabora Research Centre, Tabora, Tanzania; ^2^SACIDS Foundation for One Health, Sokoine University of Agriculture, Morogoro, Tanzania; ^3^College of Veterinary Medicine and Biomedical Sciences, Sokoine University of Agriculture, Morogoro, Tanzania; ^4^Ministry of Livestock and Fisheries, Dodoma, Tanzania; ^5^National Health Laboratory Service, Centre for Emerging Zoonotic and Parasitic Diseases, National Institute for Communicable Diseases, Sandringham, South Africa; ^6^Department of Medical Virology, Centre for Viral Zoonoses, Faculty of Health Sciences, University of Pretoria, Pretoria, South Africa; ^7^Faculty of Health Sciences, School of Pathology, University of Witwatersrand, Johannesburg, South Africa; ^8^Global Alliance for Livestock Veterinary Medicines, Edinburgh, United Kingdom

**Keywords:** Rift Valley fever virus, Clone 13 vaccine, sheep, goat, cattle, safety, immunogenicity, Tanzania

## Abstract

**Background:** Vaccination is considered to be the best approach to control Rift Valley fever (RVF) in animals and consequently in humans. This study assessed the efficacy and safety of the RVF virus (RVFV) Clone 13 vaccine under field conditions.

**Methodology:** A vaccine trial was conducted in sheep (230), goats (230), and cattle (140) in Ngorongoro district, Tanzania. Half of each of the animal species were vaccinated and the other half received the placebo. Animals were clinically monitored and bled before vaccination and at days 15, 30, 60, 180 and 360 (+/– 10) post-vaccination to measure Immunoglobulin M (IgM) and IgG antibody responses to RVFV. Survival analysis was conducted using cox-proportional hazard regression model to measure the time until an event of interest had occurred and to compare the cumulative proportion of events over time.

**Results:** Of 600 animals included in the study, 120 animals were lost during the study, leaving a total of 480 (243 in the vaccinated group and 237 in the control group) for complete follow-up sampling. There was no adverse reaction reported at the injection site of the vaccine/placebo in all animals. Abortions, deaths, or body temperature variations were not associated with vaccination (p > 0.05). By day 15 post-inoculation, the IgG seroconversion in vaccinated goats, cattle and sheep was 27.0% (*n* = 115), 20.0% (*n* = 70) and 10.4% (*n* = 115), respectively. By day 30 post-inoculation, it was 75.0% (*n* = 113), 74.1% (*n* = 112) and 57.1% (*n* = 70) in vaccinated sheep, goats and cattle, respectively. By day 60 post-inoculation, IgG seroconversion in sheep, goats and cattle was 88.1% (*n* = 109), 84.3% (*n* = 108) and 64.60% (*n* = 65), respectively. By day 180, the IgG seroconversion in sheep, goats and cattle was 88.0% (*n* = 108), 83.8% (*n* = 105) and 66.1% (*n* = 62), respectively. By day 360, the IgG seroconversion in sheep, goats and cattle was 87.2% (*n* = 94), 85.6% (*n* = 90) and 66.1% (*n* = 59), respectively. Only five animals from the vaccinated group were RVFV IgM positive, which included four sheep and a goat.

**Conclusion:** RVFV Clone 13 vaccine was well tolerated by sheep, goats, and cattle. The vaccine induced detectable, but variable levels of IgG responses, and of different duration. The vaccine is considered safe, with high immunogenicity in sheep and goats and moderate in cattle.

## Introduction

Rift Valley fever (RVF) is a climate-sensitive, economically important, an acute mosquito-borne zoonotic viral disease that is caused by RVF virus (RVFV) (1). The virus causes severe disease in livestock including cattle, sheep, goats, and camels as well as in humans mainly in Africa and the Arabian Peninsula (2). The potential for further geographical spread to RVF non-endemic areas in the world is of both veterinary and public health concern (3, 4). In livestock, the virus causes high rates of abortion (up to 100% in sheep) and high mortality rates (up to 100% in neonates) (5). In humans, the virus causes influenza-like illness and occasionally a severe disease characterized by central nervous system complications, severe jaundice, retinitis, haemorrhagic syndrome, and death may occur (6).

Between 1930 and 2007, a total of 10 RVF outbreaks were reported in Tanzania with an average inter-epidemic period of 8 years (7). The eastern Rift Valley ecosystem of the country has been identified to be at higher risk of RVF occurrence than the western Rift Valley ecosystem (7). The eastern Rift valley ecosystem is characterized by larger livestock density, predominantly impermeable soils and experiences a bimodal rainfall pattern compared with the western Rift Valley ecosystem that is characterized by lower livestock density, predominantly permeable soils, and experiences the unimodal rainfall pattern. The past RVF outbreaks in Tanzania resulted in devastating health and socio-economic consequences. The disease caused high mortality rates in domestic ruminants and humans (8). Animals lost monetary value by 34%, monthly internal market flow dropped by 37% and annual external market flow dropped by 54%. Loss due to death of domestic ruminants was > USD 6 m and the government spent about USD 4 million in the control of the disease (8).

Because RVF outbreaks typically erupt in domestic ruminants before cases are reported in humans (7, 9), their annual vaccination would be a recommended strategy to prevent the disease in animals and consequently reduce the risk of human infection. Currently, there is no licensed or commercially available RVFV vaccine for human use. The commercial vaccine that has been used against RVF in domestic ruminants in Tanzania, and other East and Southern African countries, is the live Smithburn vaccine (10–12). This vaccine requires only a single dose to induce long-lasting immunity. However, a genetic reassortment among RVFV isolates has been reported (10, 13–15). In addition, the vaccine has been associated with abortions in pregnant animals and teratogenic effects in fetuses (16). These deficiencies of the Smithburn vaccine highlight the need to develop safer and more efficacious RVFV vaccines.

As a step to address the challenges, there have been efforts to develop new vaccine candidates. One of the candidate livestock vaccines that has undergone experimental and field trials is a live RVFV Clone 13 virus that is significantly attenuated (17). It is a plaque isolate of the 74HB59 strain of RVFV recovered from a human infected with RVFV in Central Africa. In experimental challenge studies, the level of protective antibody titres induced by RVFV Clone 13 vaccine was found to be similar to those reported for the Smithburn strain, and there was no abortion or teratogeny associated with animals vaccinated with RVFV Clone 13 in contrast to those vaccinated with the Smithburn strain vaccine (18–20). Studies on the safety and immunogenicity of the vaccine in Senegal (18), South Africa (19), and Kenya (20) reported a high level of immunogenicity in domestic ruminants. In West Africa, the vaccine was reported to be well-tolerated in sheep and goats, including the pregnant ones (21). Furthermore, the vaccine did not cause detectable viremia in vaccinated ruminants, therefore minimizing the risk of vaccine virus transmission to the fetus or mosquito vectors (18). Based on these observations, it appears that the vaccine is safe in domestic ruminants when the recommended dose is administered. The RVFV Clone 13 vaccine has been registered and licensed for use in cattle, sheep and goats in South Africa, Botswana and in Namibia (10, 21).

After a vaccine has been licensed, post-registration trials need to be conducted in large populations to obtain estimates of its safety, immunogenicity and effectiveness under field conditions. We, therefore, conducted a post-licensure evaluation of the RVFV Clone 13 vaccine in the pastoral community herds to measure its safety, immunogenicity and duration of antibody persistence. Indigenous species of cattle, sheep and goats were targeted for the trial (herein also referred to as a study) in their natural environment under the traditional extensive management system characterized by nomadic pastoralism in Ngorongoro district of Tanzania.

## Materials and Methods

### Study Area

The study was conducted in Ngorongoro district in northern Tanzania. The district lies between longitude 35'30 and 36'23° E and latitude 02'45 and 4'0° S and covers an area of 14,036 km^2^. The district was purposively selected as an ideal site for this study because it has persistently reported RVF outbreaks (10 outbreaks) from 1930 to 2007 (7). The district is an integral part of the Serengeti ecosystem, which is a wildlife-livestock-human interface area. It experiences a bimodal rainfall pattern: a short rainy season from November to December, and a long rainy season between February and May. The annual precipitation ranges from 500 to 1,000 mm. The vegetation mainly consists of various shrubs and acacia bushes. The district is characterized by flat and hilly terrains interspersed with broad U-shaped valleys with predominantly impermeable soils, subject to flooding. A total of five villages, namely Pinyinyi, Engarasero, Malambo-Madukani, Malambo-Sanjani, and Malambo-Oljoro were involved.

### Source of Vaccine and Placebo

The Clone 13 RVFV vaccine and placebo (the glusamine serum diluent) were obtained from the Onderstepoort Biological Products (OBP), South Africa. On the day of vaccination, diluent was added to the bottle containing freeze-dried vaccine using sterile syringe, was mixed thoroughly until the powder was dissolved and used within two hours.

### Study Design

This was a prospective randomized double-blind vaccine-placebo trial that was implemented from July 2017 to August 2018. Based on consultations with the animal owners, sheep, goats, and cattle aged 6–24 months were targeted to facilitate retention of animals during the period of follow-up. Animals aged <6 six months were excluded from the study because they were likely to have maternally derived antibodies that could suppress vaccine-induced immune responses (22–24). Due to the lack of exact birth records, the animal's age was identified through a combination of information provided by the farmer's recall with information obtained from the dentition technique (25). In addition, to maintain compliance and response rate and minimize loss to follow-ups related to migration decisions by animal owners; female animals were oversampled within the herds as farmers reported that they were more likely to be maintained in the herd for a longer period than male animals. The number of each species of animals enrolled per herd was limited to four or six (a pair of two or three) to reduce the chances of disposal by the owners for different reasons. The strategy was also thought to simplify routine monitoring of animals by owners/herders, local veterinary officers and research team. Motivations to animal owners during the study life cycle included free veterinary consultation services and provision of prophylactic anthelminthic drugs at an interval of three months to all animals in a herd.

The animals included in the study were individually identified using ear-tags. Female animals were tested for pregnancy before been enrolled into the study. A pregnancy test in cattle was done through rectal palpation of the fetus (26). In sheep and goats, it was made through abdominal inspection and transabdominal palpation of the uterus and fetuses (27, 28). In addition, history of non-return to oestrus was sought from the owners and/herders (28). Staging of gestational age was not made. It is important to note that the techniques used for pregnancy diagnosis were considered less sensitive for the early stages of gestation and therefore chances of pregnancy misdiagnosis could not be discounted. However, as only one veterinarian was involved in the whole exercise of pregnancy diagnosis, the error was assumed to be constant in all animals. Pregnant animals were subjected to sampling frame accounting for safety of vaccine on pregnancy. The animals included in the study were those without evidence of exposure to RVFV based on pre-inoculation ELISA test results and without history of vaccination against RVF based on local veterinary records and information obtained from the owners.

### Sample Size and Sampling Strategy

Previous studies (20, 21) have shown a beneficial effect of the RVFV Clone 13 vaccine, and therefore a 1-tailed test was preferred in the estimation of the sample size. Considering a ratio of vaccinated to control animals in the trial to be 1, a precision level of 5% and power of 80% for the study to detect vaccine effect, the minimum sample size (29) required for each group (vaccinated or control) in a village was 20 cattle, 11 sheep and eight goats. This resulted in a total sample size of 78 domestic ruminants per village. A contingency of 50% was considered to account for any loss to follow-up effect resulting to a sample size of 117 that was rounded off to 120 domestic ruminants to correspond well with the number of animals targeted within herd and village. Therefore, the total sample size for the five selected villages was 600. Sheep and goats are considered more susceptible to RVFV than cattle (30), and therefore their higher numbers were sampled in each village: 46 sheep, 46 goats, and 28 cattle were included in the study.

**Figure 1:**
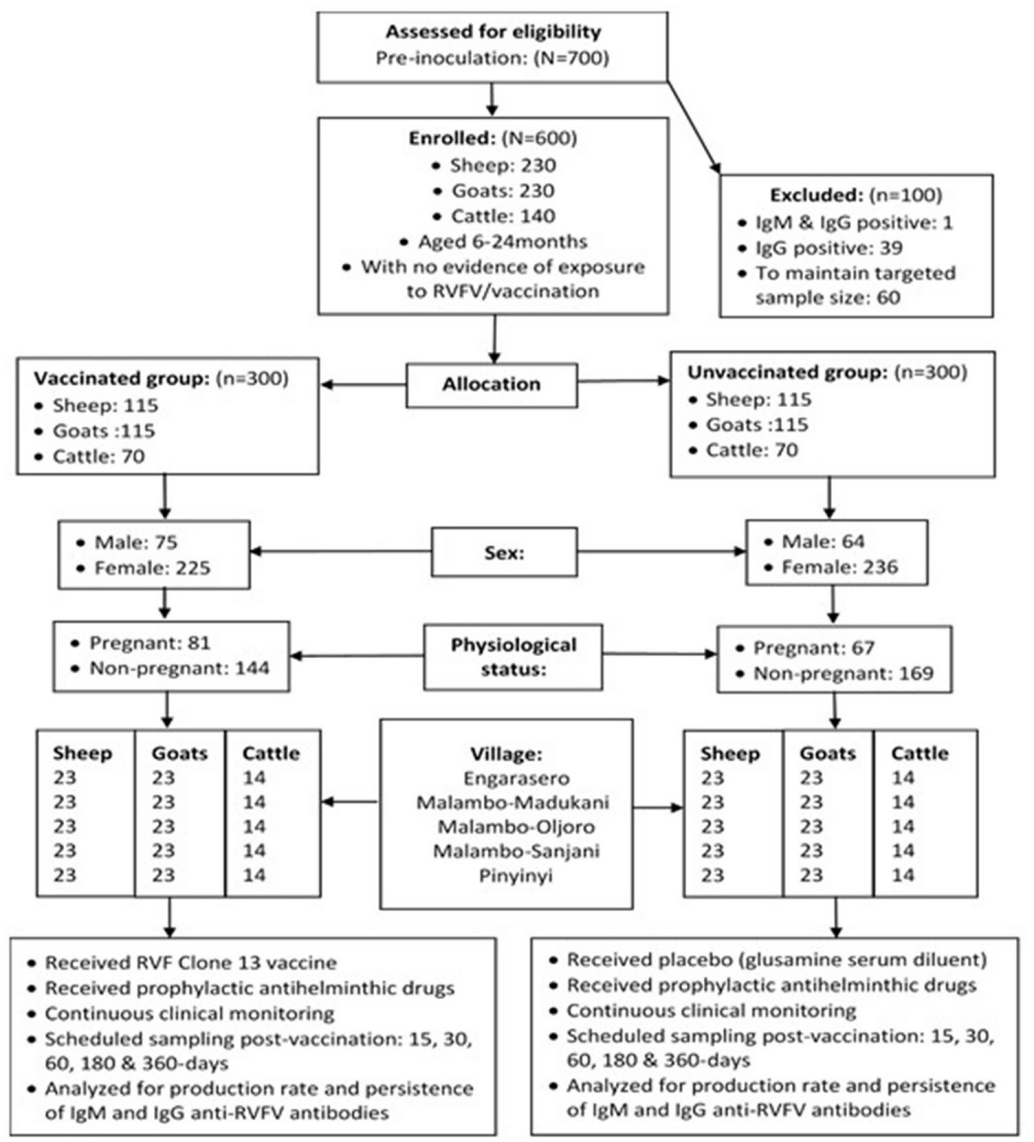
Flowchart of RVFV Clone 13 vaccine trial profile.

A total of 13 herds were initially randomly selected without replacement from the list of livestock keepers (keeping at least cattle, sheep or goats) in a village. To increase the chances of achieving the targeted overall sample size of 600 animals without evidence of previous exposure to RVFV, 700 animals were initially evaluated for the presence of antibodies to RVFV (Figure 1). When necessary, during the enrolment process, additional herds were randomly selected to obtain the required sample size for a village. To maintain the same time of observation for all animals on the effect of vaccine on parameters of interest, there was no replacement resulting from loss to follow-up animals during the study period. For the same reason, animals that aborted during the course of the study, and had not been initially enrolled as pregnant animals, were not considered in the proportional comparison analysis related to abortion between vaccinated and control animals.

### Allocation of Animals to Treatments

The study involved randomization of an equal number of animal subjects to vaccine and control (unvaccinated) groups (1:1 ratio). A total of 600 animals (sheep = 230; goats = 230; cattle = 140) were included in the study, of which more than three-quarters (76.8%) were females. Overall, allocation of animals to treatments was made such that half (300) of the overall sample size was randomly and blindly assigned to the vaccination group and the other half (300) to the placebo group (control group). An equal number of 120 animals were drawn from each of the study villages. In each village, 60 animals (sheep = 23, goats = 23 and cattle = 14) were allocated to either the vaccination or control group. At the time of enrolment, almost one-third (32%) of females (*n* = 461) were pregnant, which included 31.5% (*n* = 92), 40.4% (*n* = 94) and 35.9% (*n* = 39) of sheep, goats and cattle allocated to vaccination group and 21.7% (*n* = 92), 36.3% (*n* = 91) and 26.4% (*n* = 53) of sheep, goats and cattle, allocated to control group, respectively (Figure 1).

To maintain blinding, individuals who administered treatments (vaccine or placebo) were never involved in subsequent monitoring and clinical examination of animals and had no access to randomization and treatment records. In addition, the information on whether an animal received a vaccine or placebo was not disclosed to the owners/herders and clinical monitors. According to the vaccine manufacturer's recommendations, a single dose of 1 ml of RVFV Clone 13 vaccine or placebo was subcutaneously injected to each animal (sheep, goats or cattle) using sterile needles and syringes that were changed for each animal.

### Clinical Monitoring of Study Animals and Assessment of Vaccine Safety

Training of good clinical practices and biosafety measures was conducted to the members of the study team before its implementation. The vaccine was stored in the refrigerator (4–8°C) in a laboratory at the Sokoine University of Agriculture, and was maintained in the same temperature range in a portable electrical refrigerator operated in a vehicle during the entire period of transportation and use in the field, as per manufacturer instructions. Animal owners were advised to continue with their usual animal husbandry practices as it was before enrolling animals into the study. They were also requested not to discriminate or treat differently the animals in the trial from the rest of the group in their usual herd management practices.

The animals were not confined post-inoculation but were left in their natural environment characterized by nomadic pastoralism so that the vaccinated and control groups could have similar levels of natural exposure to all known and unknown confounding risk factors. As the animals were traditionally trekked long distances in search of pasture and water during periods of drought, in each participating herd, two members of the animals owning family were trained to observe animals routinely. They were provided with forms with an identification number for each animal to fill in the health-related events including births, abortions, illnesses, mortalities and other clinical manifestations/events. They were provided with phone numbers for the local veterinary officers and the research coordinator to enhance two-way communication. The livestock field officers in the study areas served as the frontline workforce to clinically examine the animals for any reactions at the site of injection and other clinical manifestations. They also maintained active consultations with animal owners and the study coordinator to address any resulting challenges.

Blood sampling was conducted prior to inoculation (day 0) and on days 15, 30, 60, 180, and 360 (+/– 10) post-inoculation. Repeated sampling and examination of blood samples were conducted to assess the proportion of immunological response and persistence of produced antibodies throughout the study period. Blood samples were collected aseptically by jugular vein puncture into labeled plain vacutainer tubes. They were kept in a cool box with ice packs before separating the serum from coagulated whole blood into labeled 1.8 ml leak-proof CryoVial^®^ tubes. They were then stored in −196°C liquid nitrogen gas in the field before been transferred to the laboratory, where they were kept in a freezer at −20°C until analysis.

### Measurement of Safety, Immunogenicity and Antibody Persistence

All animals were monitored for adverse effects during the follow-up period. The occurrence of illnesses and deaths were monitored in all animals and the occurrence of abortion was observed in females. The rectal temperature of each animal was recorded prior to vaccination (day 0), during the first three days' post-inoculation, and then during the scheduled blood sampling visits on days 15, 30, 60,180, and 360-post vaccination. The collected parameters were compared between pre- and post-vaccination.

The production and persistence of immunoglobulin M (IgM) and immunoglobulin G (IgG) antibodies against RVFV were determined by testing the collected serum samples. This was carried out using commercial ID Screen^®^ RVFV competitive multi-species ELISA (ID Vet, France) and anti-RVFV IgM using ID Screen^®^ Rift Valley fever IgM capture ELISA (ID Vet) according to the manufacturer's instructions (ID-VET, Innovative Diagnostics, Grabels, France).

### Statistical Analysis

The clinical data and laboratory results were entered into a Microsoft Excel spreadsheet, cleaned and coded before been imported into STATA version 13.1 (Statacorp, College Station, TX, USA) for descriptive statistical analysis. The time-probability of producing anti-RVF antibodies (animal-days) contributed by each animal was calculated using life table analysis (31). The outcome of interest was anti-RVFV antibody production rate in vaccinated and control animals, which was expressed per 1,000 animal-days. Exit from the study was due to loss to follow-up (death, predation or sold out) or end of the follow-up period. The Chi-square test produced from a log-rank test of equality was used to compare antibody production rates between vaccinated and control groups. The null hypothesis tested was that there was no difference regarding the rate of antibody production between the two groups.

We considered potential covariates on the rate of anti-RVFV antibodies production, which included animal species, sex, age group, pregnancy status, and village. To take into consideration time until an event of interest had occurred and compare the cumulative proportion of events over time, while adjusting for potential influential covariates, survival analysis was conducted using cox-proportional hazard regression model, utilizing both univariable and multivariable time-dependent regression modeling approaches (31, 32). The term hazard in this study refers to the probability that an individual animal under observation at time t had an event defined as anti-RVFV antibody at that time. Plotting this instantaneous hazard against time provided the hazard function and the rate at which the instantaneous rate of anti-RVFV antibody production changed with respect to time, which was used to estimate the rate ratio of antibody production between vaccinated and control groups.

As all the covariates in the data set were considered relevant to the model, initial screening in the univariable analysis was made at a cut-off *p* ≤ 0.20. The final evaluation of covariates was made in the multivariable model utilizing the likelihood ratio statistic and a *p* ≤ 0.05. The variables included in the final model were limited to those that did not show significant collinearity. Interaction terms were then introduced into the model to examine the potential presence of effect modification. Herd was treated as a random effect variable to account for possible variation attributed by individual herds. Village was treated as fixed effect variables to account for possible correlations attributed by individual villages. The non-parametric method of Kaplan Meier survival curves and semi-parametric proportional hazard models were used to explore the determinants of time to sero-conversion attributed by vaccine and as a result of other covariates (32).

### Ethical Consideration

The study was approved by the Directorate of Veterinary Services of the Ministry of Livestock and Fisheries in Tanzania (Ref. No. PA116/187/73). Permission to conduct the study in Ngorongoro district was provided by the District Executive Director Office. Consultative meetings were held with the village leaders, Livestock Field Officers and animal owners to introduce the study and agree on the implementation plan. Permission to include animals in the study was sought from the owners.

## Results

### General Observations

A total of 600 (vaccine group: 300 and control group: 300) clinically health animals from 65 herds (13 herds from each village) were enrolled into the study. They included sheep (230), goats (230), and cattle (140) representing an equal number of 120 animals drawn from Engarasero, Malambo-Madukani, Malambo-Oljoro, Malambo-Sanjani, and Pinyinyi villages of Ngorongoro district. Females accounted for over three-quarter (76.8%) of all animals, and about an equal proportion of them (48.8%, *n* = 461) were allocated to vaccine (48.8%) and control (51.2%) groups (p > 0.05). A relatively higher proportion of males (54.0%, *n* = 139) were allocated to vaccine group and 46.0% of them to control group, although the allocation difference was not statistically significant (*p* > 0.05). Almost one-third (32.1%) of females were pregnant prior to inoculation, which included 29, 38, and 14 sheep, goats, and cattle in the vaccinated group and 20, 33, and 14 sheep, goats, and cattle in the control group. The baseline body temperature values (recorded prior to inoculation) for animals allocated to vaccine and control groups ranged from 36.6 to 39.1°C (median = 37.9°C) and 36.9 to 39.1°C (median = 37.9°C), respectively with no evidence of statistical difference between the two groups (*p* > 0.05). Overall, a total of 120 (20%) animals were lost during the follow-up period. This included 57 (19.0) from vaccinated group and 63 (21.0%) control group. Thus, complete follow-up data was available for 480 (vaccinated = 243; control = 237). The reasons for loss to follow-up (*n* = 120) were death of unknown cause (77.5%), predation by wild animals (18%) and been sold out (7.5%) (Table 1).

**Table 1:** Distribution of loss to follow-up of animals and associated reasons during the entire study period.

**Animal**	**No. enrolled**	**No. death**	**Sold out**	**Predation by wild animals**	**Total lost (%)**
**Sheep**
Vaccinated	115	15	1	1	17 (14.8)
Control	115	17	4	3	24 (20.9)
**Goats**
Vaccinated	115	16	3	5	24 (20.9)
Control	115	22	1	7	30 (26.1)
**Cattle**
Vaccinated	70	14		2	16 (22.9)
Control	70	9		0	9 (12.9)
**Total vaccinated**	300	45	4	8	57 (19.0)
**Total control**	300	48	5	10	63 (21.0)
**Total overall**	600	93	9	18	120 (20)

### Vaccine Safety

**Figure 2:**
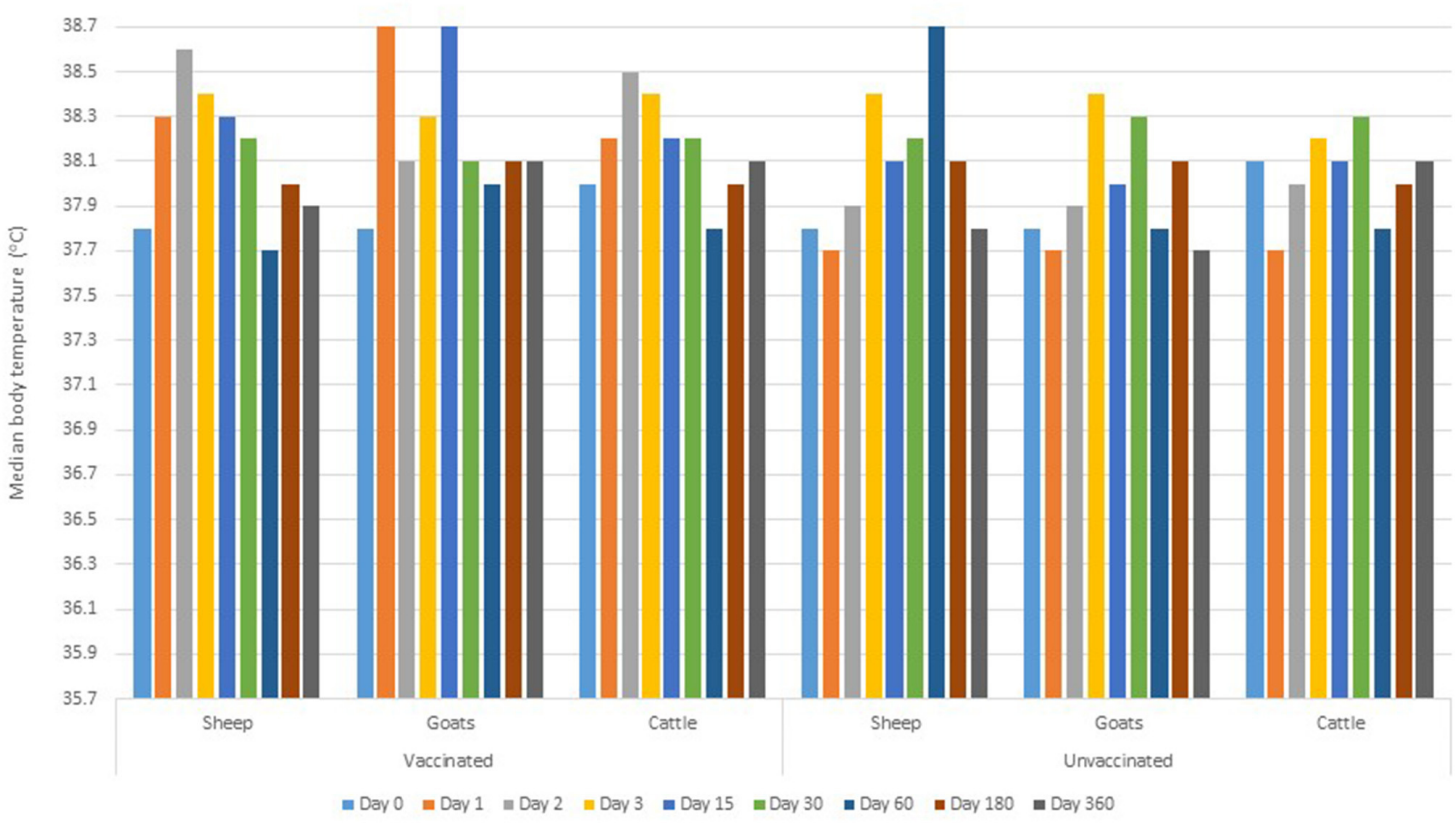
Median body temperature during the pre-inoculation (day 0) and post-inoculation days.

There was no adverse reaction reported at the injection site of vaccine/placebo in all animals. There was no statistically significant difference in the baseline (prior to inoculation) body temperature readings between vaccinated and control groups (*p* > 0.05). Comparing the post-inoculation between-group body temperature, the parameters (for all animal species) were significantly higher in vaccinated group than control group at day 1 and 2 (*p* = 0.001). At day 15 post-inoculation, the body temperature in vaccinated goats was significantly higher than in the control group (*p* = 0.025). There was no statistical evidence on the variation of body temperature readings between vaccinated and control groups for the rest of the clinical monitoring intervals (Figure 2).

**Figure 3:**
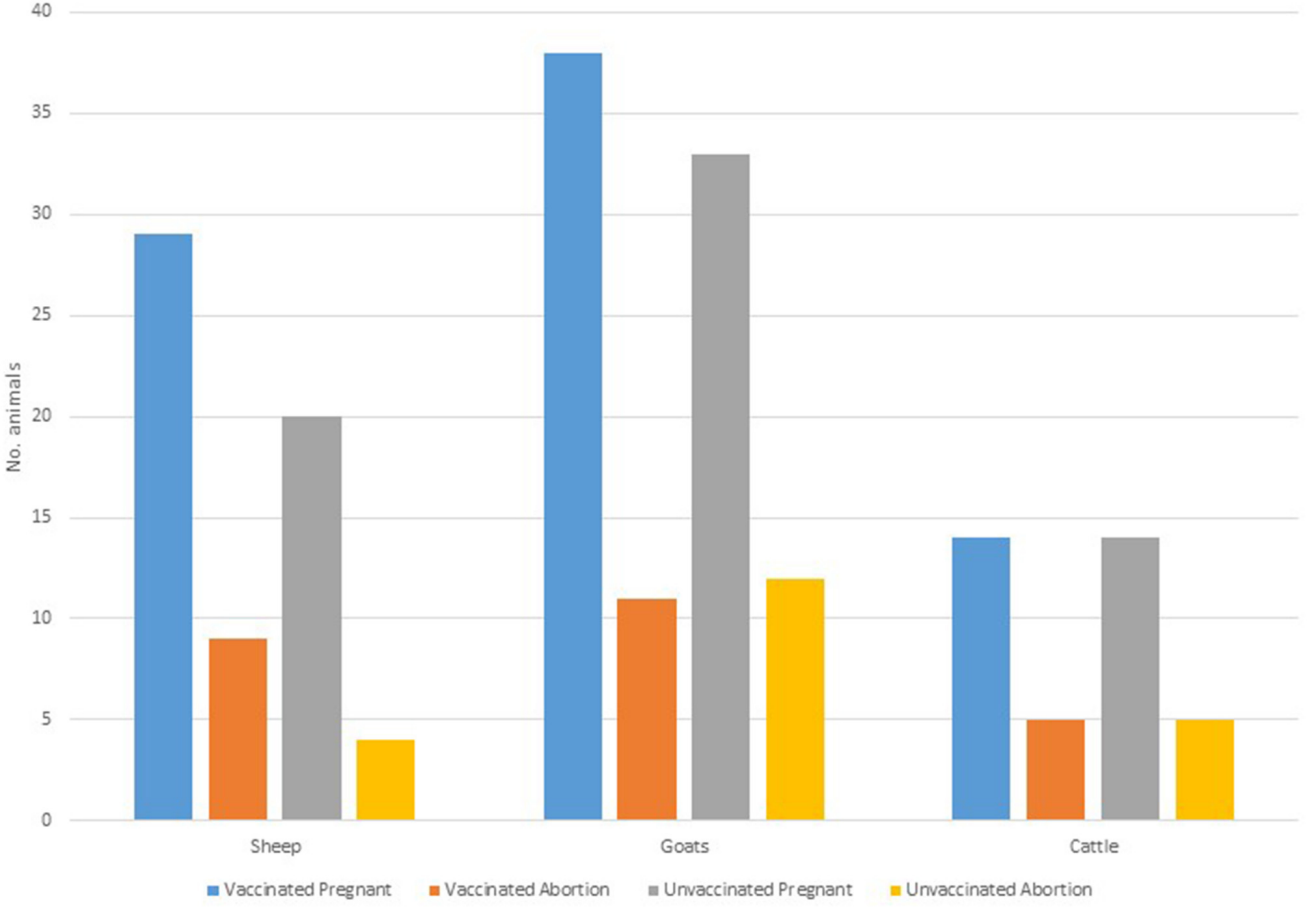
Distribution of abortion in vaccinated and control groups stratified by animal species.

**Figure 4:**
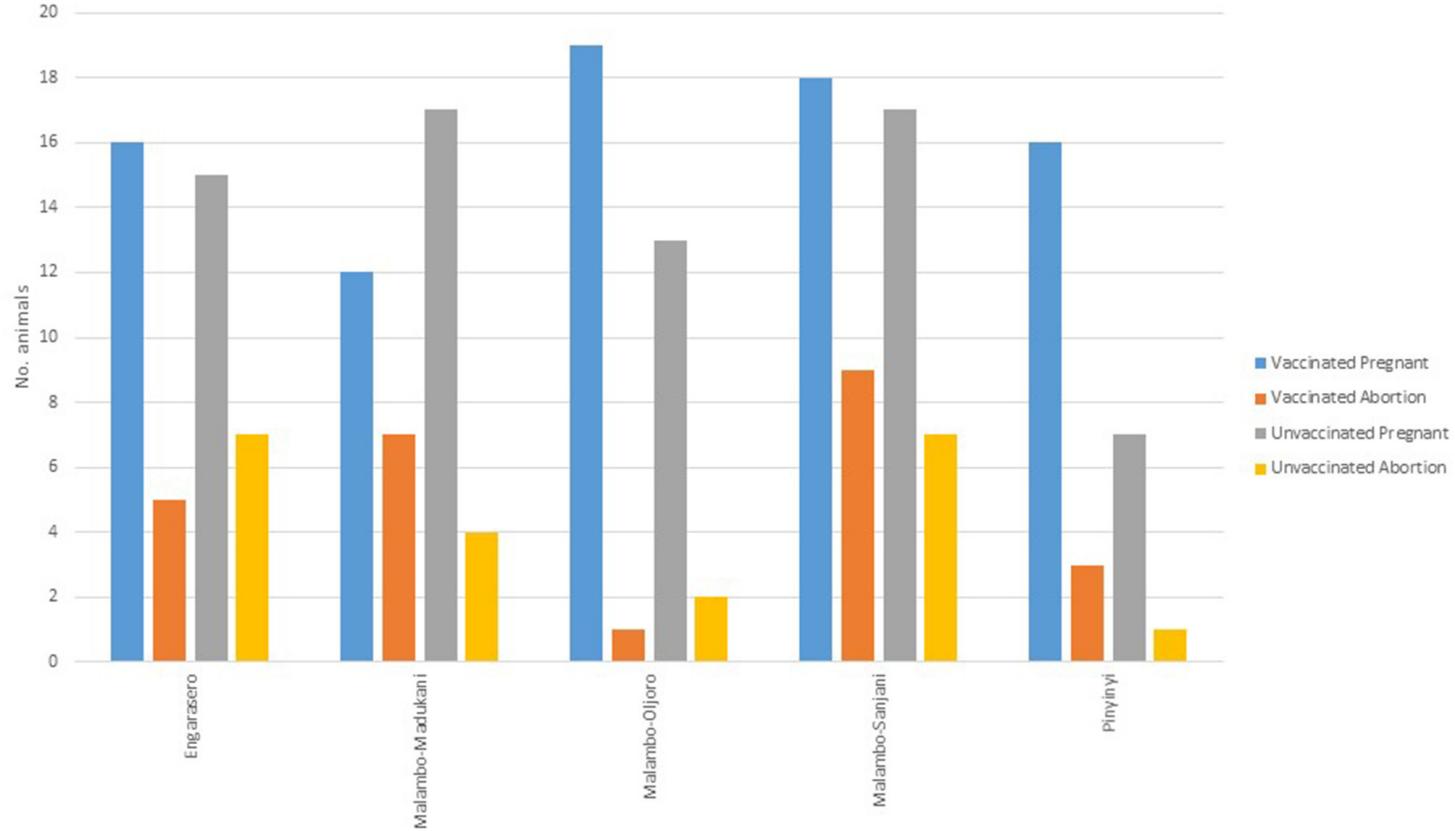
Distribution of abortion in vaccinated and control animal groups stratified by villages.

Of 148 animals that were pregnant, a 68.9% (vaccinated = 69.1%, *n* = 81; control =68.7%, *n* = 67), carried their pregnancies to term. A total of 46 (vaccinated = 25; control = 21) animals aborted (specific causes of abortions were not established). Within the species, the highest proportion of abortion was observed in cattle (35.7%, *n* = 28), followed by goats (32.4%, *n* = 71) and sheep (26.5%, *n* = 49) (Figure 3). The highest proportion of abortion was recorded in Engarasero (38.7%, *n* = 31), Malambo-Madukani (37.9%, *n* = 29), and Malambo-Oljoro (34.4%, *n* = 32) (Figure 4). There was, however, no significant difference in the rate of abortion between the groups, species or villages (*p* > 0.05). Similarly, increased body temperature was not associated with events of abortion (p > 0.05).

A total of 93 animals died, 18 were consumed by wild animals, and nine were sold out. Of the animals that died, 45 (48.4%) were from the vaccinated group and 48 (51.6%) from the control group, with no evidence of a statistically significant difference between the two groups (*p* > 0.05). Likewise, there was no evidence of statistically significant difference in the rate of death between females (17.4%, *n* = 438) and males (12.6%, *n* = 135); 6–12 months age group (13.7%, *n* = 284) and 13–24 months age group (18.7%, *n* = 289); or animal species sheep (18.6%, *n* = 221), goats (15.0%, *n* = 214), and cattle (14.5%, *n* = 138) (*p* > 0.05). Of 18 animals consumed by wild animals, eight (44.4%) were from the vaccinated group and 10 (55.6%) were from the control group. Of nine animals that were sold out, four (44.4%) were from the vaccinated group and five (55.6%) were from the control group. The chi-square test suggested no statistical difference for animal deaths, predation by wild animals or been sold out between vaccinated and control groups (*p* > 0.05). Based on these observations, none of these events (deaths, abortion, predation or been sold out) were judged to be related to vaccination. Clinical manifestations recorded prior to animal deaths included circling, unbalanced gait, gait impairments, movement disorders, and weakness of limbs. During the follow-up period, there was no clinical form of RVF observed in the study areas.

### Survival Analysis of Time to Anti-RVFV Antibodies Production

**Figure 5:**
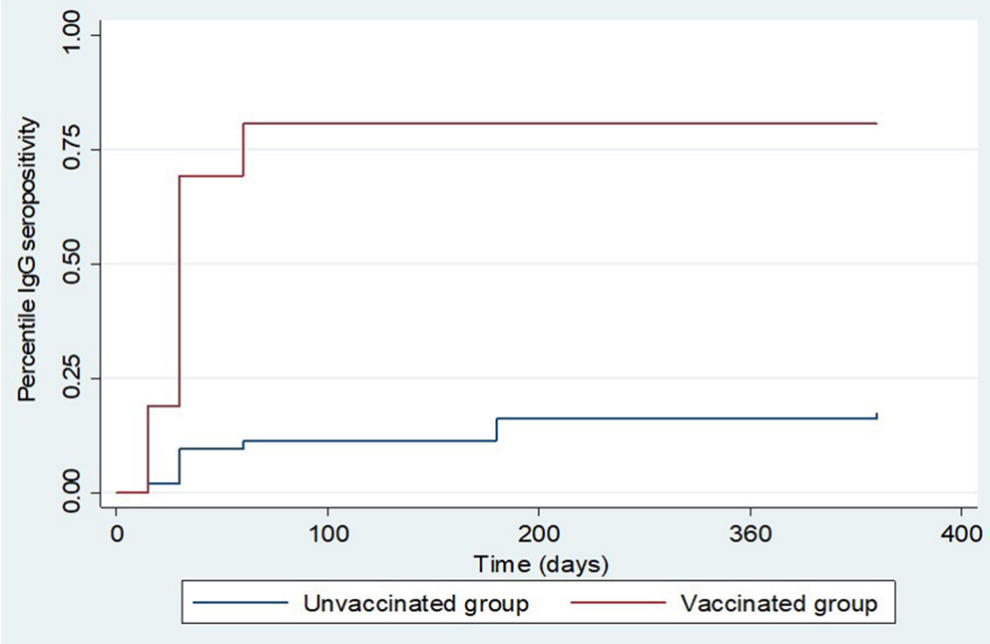
Kaplan Meier survival curves showing overall IgG production estimates in the vaccinated and control groups.

**Figure 6:**
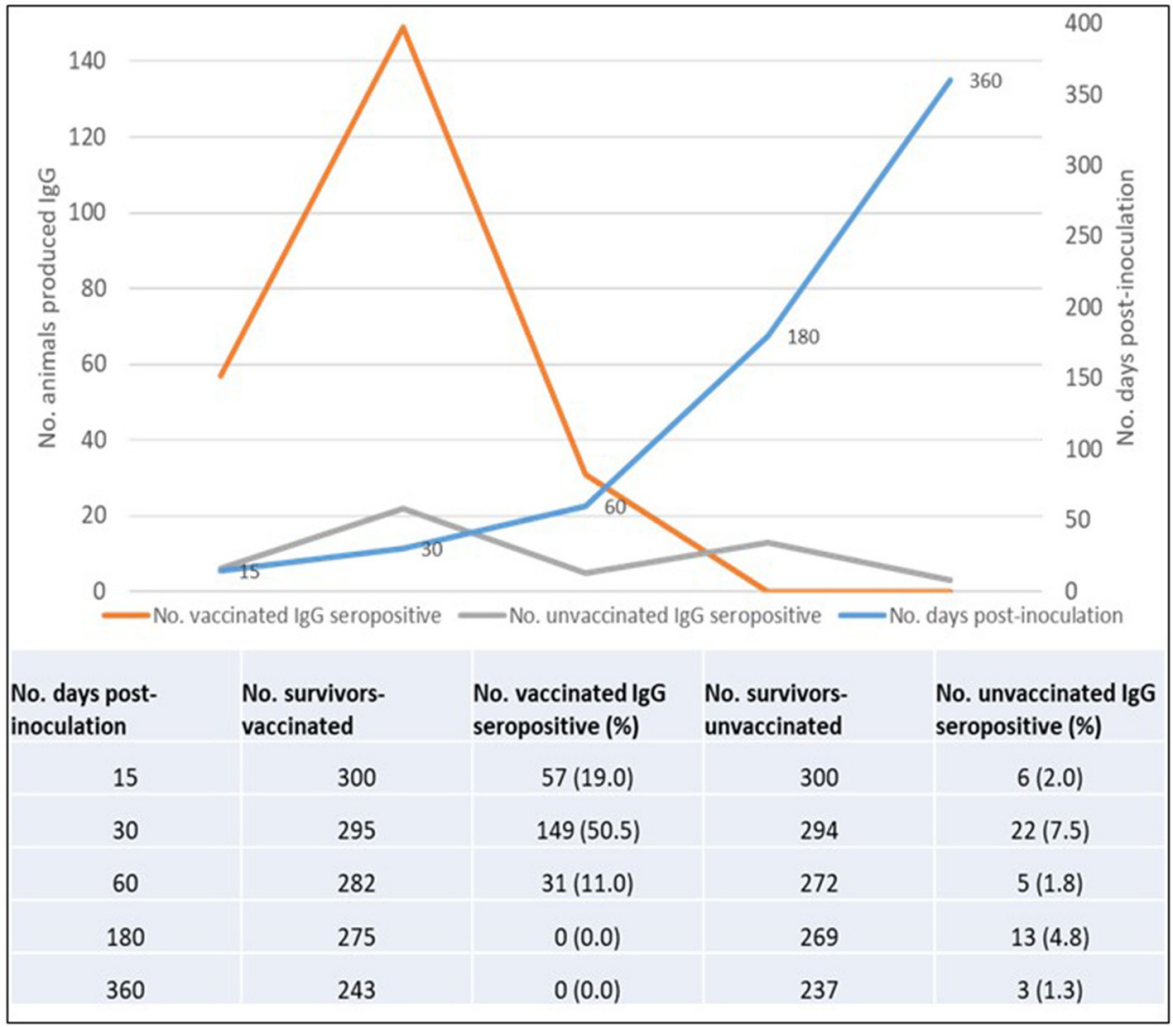
Overall IgG seropositivity rate in vaccinated and control animals.

The overall total animal-days of producing IgG anti-RVFV antibodies for animals from both the vaccinated and control groups was 107,490 with an IgG seropositive value of 286 translating to an overall IgG production rate of 2.66 cases per 1,000 animal-days (CI: 2.37–2.99). For the vaccinated group, the total animal-time was 24,840 animal-days translating to IgG production rate of 9.5 cases per 1,000 animal-days (CI: 8.40–10.84), and for the control group, it was 82,650 animal-days with IgG production rate of 0.6 cases per 1,000 animal-days (CI: 0.45–0.78). Kaplan-Meier curve showed an apparent increased difference in the rate of anti-RVFV IgG seroconversion in favor of the animals in the vaccinated group against those from the control group. By day 15 post-vaccination, 19.0 and 2.0% of animals from vaccinated and control groups, respectively, had IgG seroconversion. The percentiles of the rate of IgG production derived from the curve indicated that over 50 and 75% of animals in the vaccinated group had produced IgG by day 30 and 60 post-inoculation, respectively. Over the observed range of data (up to 360 days), the probability of IgG production in the control group did not reach 25% (Figure 5). Overall, the vaccinated animals accounted for 82.9% (*n* = 286) of animals that produced IgG antibodies post-inoculation (*p* < 0.001). By the end of day 360 of the study, 237 (79.0%) and 49 (16.3%) animals in the vaccinated and control groups seroconverted, respectively. Peak IgG seropositivity was detected 30 days' post-inoculation in vaccinated (149/295) and control (22/294) groups. Beyond day 60 post-inoculation, there was no additional seropositivity detected in the vaccinated group. In the control group, an additional 13 and 3 IgG seropositivity was detected on days 180 and 360 post-inoculation, respectively (Figure 6).

We observed that once produced, all surviving animals maintained detectable IgG to the end of the follow-up period. However, we did not measure the level of antibody titer produced over time. The log-rank test for equality of survivor functions indicated strong evidence of statistical difference in the rate of IgG production between animals in the vaccinated and control groups (*p* < 0.0001). A significantly higher proportion of IgG seropositivity was consistently observed in animals from the vaccinated group than those from the control group throughout the follow-up period (Figure 6). During the follow-up period, five animals from the vaccinated group produced IgM anti-RVFV antibodies, which included a sheep, a goat (detected 15 days post-inoculation), and three sheep (detected 30 days post-inoculation). There was no evidence of detectable IgM in the subsequent samples taken in the vaccinated group from day 60 to day 360. The IgM antibodies were not detected in any of the animals from the control group.

**Figure 7:**
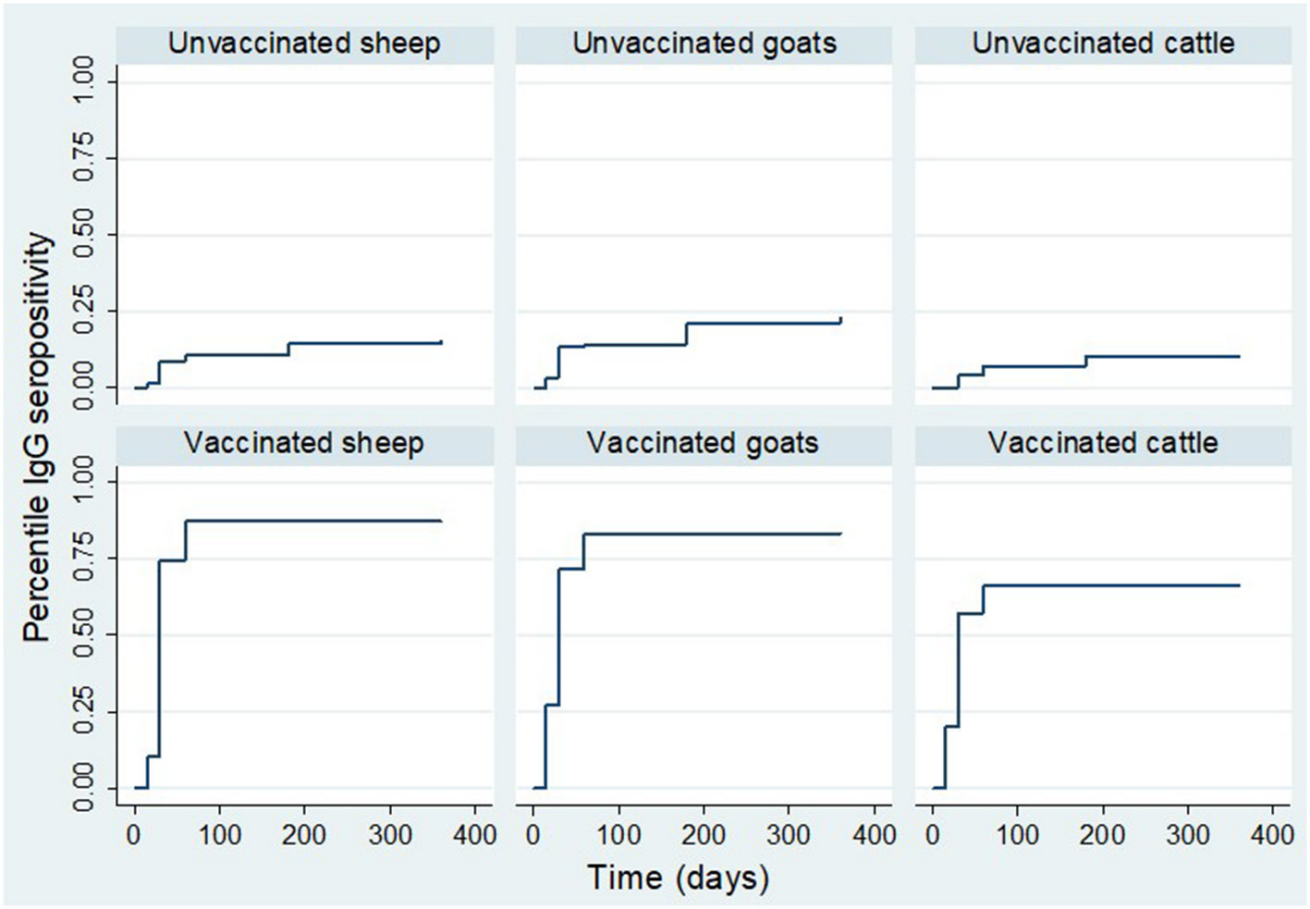
Kaplan Meier survival curves showing IgG production estimates in each treatment group stratified by animal species.

The cumulative proportion of animals that seroconverted is reported against the total cumulative number of animals that survived in the respective groups by the time of sampling. Analysis of IgG seropositivity between animal species indicated that for vaccinated animals; during the first 15 days' post-inoculation, a higher rate of IgG seropositivity was observed in goats and cattle than sheep. During this period, the IgG seroconversion in goats, cattle and sheep was 27.0% (*n* = 115), 20.0% (*n* = 70) and 10.4% (*n* = 115), respectively. By day 30 post-inoculation, it was 75.0% (*n* = 113), 74.1% (*n* = 112) and 57.1% (*n* = 70) in vaccinated sheep, goats and cattle, respectively. By day 60 post-inoculation, IgG seroconversion in sheep, goats and, cattle was 88.1% (*n* = 109), 84.3% (*n* = 108) and 64.60% (*n* = 65), (Figure 7).

For the control group, by day 15, the IgG seroconversion observed in sheep and goats was 1.7% (*n* = 115) and 3.5% (*n* = 115), respectively. There was no seroconversion observed in cattle during the period. By day 30, the cumulative seroconversion of IgG was 13.5% (*n* = 111), 8.9% (*n* = 113) and 4.3% (*n* = 70) in goats, sheep and cattle, respectively. The IgG seroconversion by day 60 was 14.3% (*n* = 105), 8.7% (*n* = 103) and 6.3% (*n* = 64), respectively. By day 180, it was 21.4% (*n* = 103), 12.8% (*n* = 102) and 9.4% (*n* = 64), in goats, sheep, and cattle, respectively. By day 360, it was 20.7% (*n* = 92), 9.3% (*n* = 86) and 8.5% (*n* = 59) in goats, sheep and cattle (Figure 7).

**Figure 8:**
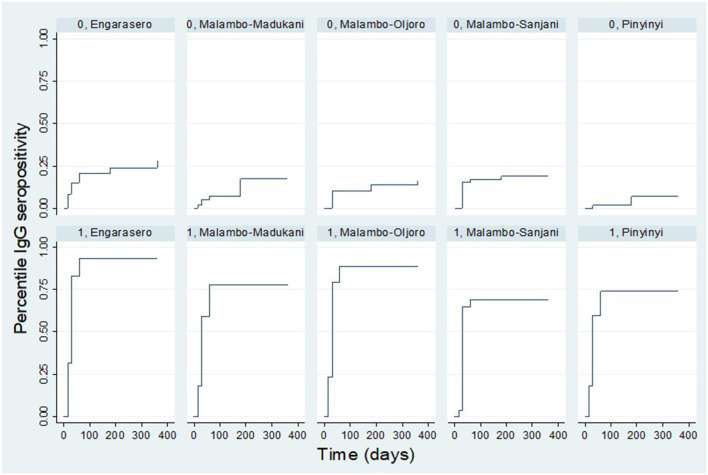
Kaplan Meier survival curves showing IgG seropositivity rate in each treatment group stratified by village. “0” and “1” denote control and vaccinated groups, respectively.

The results show further that the IgG seropositivity rate varied by village. For vaccinated animals, a higher rate of seropositivity was observed in Engarasero, Malambo-Oljoro, and Malambo-Madukani. By day 60 post-inoculation, over 75.0% of vaccinated animals in these villages were IgG seropositive and there was no seropositivity detected beyond this period. For animals from the control group; a higher rate of IgG seropositivity was observed in Engarasero (20.0%) and Malambo-Sanjani (16.0%) by day 60 post-inoculation. Contrary to the vaccinated group, IgG seropositivity was detected in control animals beyond day 60 of observation. By 180 days, the seropositivity rate in Engarasero, Malambo-Sanjani, Malambo-Madukani, Malambo-Oljoro, and Pinyinyi increased to 23.0, 18.0, 15.0, 13.0 and 7.0%, respectively. Afterward, by end of the follow-up period seropositivity was detected only in Engarasero mounting to 27.0 and 15.0%, respectively (Figure 8).

### Cox Proportional-Hazard Regression Analysis

The results of Cox proportional-hazard univariable regression analysis for time-to-event showed that at any time during the follow-up period, vaccinated animals were 7.2 times as likely to produce IgG compared to those from the control group (CI: 5.25–9.84). Sheep and goats were 35% (CI: 0.98–1.87) and 49% (CI: 1.08–2.07), respectively, more likely to develop IgG over a shorter period of time than cattle. However, there was insufficient statistical evidence for the rate in sheep to vary from that in cattle. Compared with animals in Pinyinyi, those in (respective likelihood percentage and associated 95% confidence intervals in parentheses) Engarasero (73%, CI: 1.19–2.50), Malambo-Madukani (23%, CI: 0.83–1.81), Malambo-Oljoro (38%, CI: 0.94–2.02), and Malambo-Sanjani (9%, CI: 0.73–1.61) were likely to develop IgG over a shorter period, with statistically significance difference been observed in Engarasero. There was weak statistical evidence for an observed chance of 16% in female animals to develop IgG faster than males (CI: 0.88–1.54). Likewise, weak statistical evidence was observed for an older animal's chance of 21% to develop IgG over a shorter period than its younger counterparts (CI: 0.96–1.53). Being pregnant was significantly associated with an increased rate of immunogenicity. A pregnant animal was 45% more likely to develop IgG over a shorter period compared to a non-pregnant one (CI: 1.11–1.90).

### Multivariable Proportional-Hazard Cox Regression Model

The final multivariable Cox regression model indicated that the rate of IgG seropositivity in an animal vaccinated with RVFV Clone 13 vaccine was 7.52 fold the rate in the control animal (CI: 5.48–10.31). The model shows the dependence of anti-RVF IgG antibody production rate after vaccination with RVFV Clone 13 vaccine on covariates. The vaccine was more immunogenic in goats and sheep than cattle. Compared with cattle, the rate of IgG seropositivity was 59.0% higher in goats (CI: 1.15–2.20) and 35.0% higher in sheep (CI: 0.97–1.87), however with insufficient statistical evidence in the latter. Animals in Engarasero and Malambo-Oljoro had a significantly higher rate of IgG seropositivity of 89.0% (CI: 1.31–2.73) and 47.0% (CI: 1.00–2.15) than animals in Pinyinyi. Although no significant difference was apparent, animals in Malambo-Madukani and Malambo-Sanjani had 21.0% (CI: 0.82–1.78) and 2.0% (CI: 0.69–1.52) higher rates of IgG seropositivity than those in Pinyinyi (Table 2). The likelihood chi-square test suggested that the model with inoculation groups, animal species and village fitted the data well (*p* < 0.0001).

**Table 2:** A multivariate analysis of relationship between animal-group, animal-species, animal-village, and IgG sero-conversion.

**Variable**	**Rate of IgG production**	**95% CI**	***P*-value**
**Group**
Control	reference group		
Vaccinated	7.52	5.48–10.31	<0.001
**Animal**
Cattle	reference group		
Sheep	1.35	0.97–1.87	0.075
Goats	1.59	1.15–2.20	0.005
**Village**
Pinyinyi	reference group		
Engarasero	1.89	1.31–2.73	0.001
Malambo-Madukani	1.21	0.82–1.78	0.344
Malambo-Oljoro	1.47	1.00–2.15	0.049
Malambo-Sanjani	1.02	0.69–1.52	0.914

## Discussion

The RVFV Clone 13 vaccine had undergone testing under experimental conditions with limited studies under field conditions (18–20). Our study was designed to demonstrate safety, immunogenicity and antibody responses following vaccination with RVFV Clone 13 vaccine in domestic ruminants kept by nomadic pastoralists in the natural environment and under the traditional management system. The primary endpoint was to assess the proportion of animals producing antibodies against RVFV over time. In the absence of the disease outbreak during the study period and the lack of recent RVF vaccination history, we attempt to translate the results to infer vaccine effectiveness indirectly. However, the observed evidence of seroconversion in control animals, and RVF outbreaks reported in the study area (7), highlighted important limitations related to our study.

During the follow-up period, there were no adverse effects associated with inoculations suggesting that the RVFV Clone 13 vaccine and placebo were well-tolerated by domestic ruminants. This observation collaborates with findings from a previous study that found that the vaccine does not cause local or systemic reactions (33). The body temperature in vaccinated sheep, goats, and cattle during days 1 and 2, and in goats on day 15 post-inoculation was higher than in the animals from the control group. It is likely that subcutaneous vaccination induced transient viraemia (33) or rather was a variation in the function of an individual animal's body in responding to the inoculation. Lack of significant difference related to body temperature, abortions or deaths between vaccinated and control groups in our study, supports the previous findings that the vaccine is safe and efficacious in domestic ruminants (18–21). Although we did not confirm the causes of deaths, the clinical manifestations developed before death were consistent with a neurological disease of sheep and goats, known locally as “*ormilo”* (34). The disease has been reported as the leading animal disease of concern amongst the Maasai pastoralists in the study area (34). Other common disease conditions in the study area to be considered in the differential diagnosis would include bacterial infections such as brucellosis (35).

The serological analysis indicated that the vaccine was effective in inducing the production of anti-RVFV IgG antibodies in domestic ruminants, but with different rates of immunogenicity. The higher rate of IgG production observed in sheep and goats over that of cattle supports the findings from other studies (20, 36). We administered the same vaccine dose of 1 ml for sheep, goats, and cattle as per the manufacturer's recommendation. It is not known if administering a higher vaccine dose in cattle would trigger a different immunological response. The results of our study showed that once developed, the IgG seropositivity was maintained in animals throughout the follow-up period, which is consistent with the protective period defined by the vaccine manufacturer. The findings are comparable with those reported elsewhere (10, 18–21). A study in Kenya (20) found that apart from goats, almost half of vaccinated sheep and cattle failed to maintain developed IgG to the end of a 12-month vaccine trial follow-up, which contradicts our findings. However, a vaccine trial in Senegal reported maintenance of produced IgG in sheep and goats vaccinated with RVFV Clone 13 during the 12-month follow-up, which is similar to our findings. The differences in the experimental set-up, number of experimental units, study areas, age structure and breeds of animals involved, variations in the endemicity of RVF in the areas, vaccine batches used and the ELISA method used may partly explain the observed discrepancy/similarities between the study findings.

The RVFV Clone 13 vaccine-elicited robust IgG antibody response at the rate of almost eight times in vaccinated against control animals. Considering the seropositivity as a function of the proportion vaccinated, the proportion that seroconverted post-vaccination, and the proportion of seropositivity following natural infection (control animals); we translate the observed rate of anti-RVFV production in vaccinated vs. control animals as a correlate of the protective magnitude of the vaccine against RVF, under the assumption that seropositivity implies protection (17–20). Further analysis of the effectiveness of the vaccine during expected periods of the disease epidemic will generate additional data.

The peak timing of IgG production after vaccination with RVFV Clone 13 makes robust estimation of the strategic period of vaccination particularly important. Analysis of the dynamics of anti-RVFV IgG antibodies after vaccination reached its peak on day 30 post-inoculation with half of the vaccinated animals expressing immune response. This observation provides biologically relevant information on vaccine-induced immune responses suggesting that the onset of vaccination programmes should be initiated at least 30 days before the expected period of the disease epidemic.

Although IgG production rate was comparatively higher in vaccinated than control animals, it remains unclear on the potential drivers of a concordance peak of antibody production observed by day 30 post-inoculation in the two groups. It should be however noted that the study was carried out in an area with a history of RVF outbreaks, which may partly explain the antibody productions dynamics observed in vaccinated and control animals. We cannot underestimate the potential transmission of wild viruses at the same time in vaccinated and not vaccinated animals.

The rate of IgG production in vaccinated animals followed a biphasic pattern, with a rapid increase to its peak within the first 30 days followed by a decline by day 60 post-inoculation. By contrast, the rate in the control group followed a polyphasic pattern with an increased rate during the first 30 days, followed by a decline by day 60, a slight increase by 180 days with a further decline by 360 days. The polyphasic pattern of the proportion of IgG seropositivity observed in the control group presumably supports the concept of endemicity of the disease in the study area. In this study, we assumed that anti-RVFV antibodies detected in control animals was a result of a natural infection virus challenge that was considered to be mild as there were no clinical manifestations suggestive of the disease observed during the follow-up period. In the disease-endemic areas, it is not uncommon for animals to carry asymptomatic infections with RVFV (20). The possibility of RVFV transmission from vaccinated to control animals remains unclear and provides a potential limit in our data analysis and translation. A similar consideration for the possibility of RVFV Clone 13 transmission within the herds was made by Njenga and colleagues (20). This notion seems, however unlikely based on the results from a study conducted by Makoschey and colleagues (33). The authors reported that the Clone 13 vaccine was safe in young lambs, even after multiple administrations of an overdose *via* different inoculation routes, and that the vaccine did not spread to the environment or to other animals, and did not revert to virulence. Although there is no robust data available, a previous study raised concerns about the possibility of genetic reassortment between S segment in the Clone13 vaccine and virulent strains in the field (37).

The results of the multivariable Cox regression model suggested that the rate of immunological response to RVFV following vaccination was not sex, age or pregnancy-dependent, rather the model suggested dependence on animal species and village as covariates. Animals in Engarasero, Malambo-Madukani, Malambo-Oljoro, and Malambo-Sanjani had higher rates of IgG production than those in Pinyinyi. The observed results were unexpected because contrary to the rest of the study villages, Pinyinyi is in the proximity of Lake Natron and characterized by an irrigation scheme for agricultural production making it a hypothetically suitable habitat for RVF vector activity throughout the year, which is against a consideration being made for other study villages. Our results show that control animals in Pinyinyi expressed the lowest rate of IgG production, suggesting low natural RVFV infection dynamics in this area. We however cannot account for a possibility of animals from the study villages mixing, especially during the dry season when animals were moved long distances in search of pasture and water, and the effect of the practice on the study findings.

This study found a low rate of detectable anti-RVFV IgM antibodies, as only five vaccinated animals (sheep and goats) showed IgM serological evidence over the follow-up period. It remains unclear why the majority of vaccinated sheep and goats, and all cattle failed to produce detectable IgM antibodies. This observation contrasts the findings from studies in Kenya (20) that reported cattle vaccinated with RVFV Clone 13 failing to develop anti-RVFV virus IgM antibodies and a study in Senegal (21) that reported a very low proportion of IgM seropositivity in vaccinated sheep and goats. The IgM antibodies produced in our study were short-lived and waned over time as there was no evidence of detectable levels beyond day 30 post-vaccination. It has been established both in infection and in vaccination studies that IgM antibodies last only for up to 60 days post-infection (38) or even persisting up to 150 days post-infection (39). Other studies have shown that anti-RVFV IgM antibodies were lost in 50% of animals by 45 days after infection and were absent after two or three (40) or four (41) months after infection. A relatively shorter sampling interval than the one used in our study would likely provide a better understanding of antibody production and persistence dynamics.

There are important limitations worth noting in this study. The study animals were left in their natural environment after inoculation. During the dry season, it remained a common practice for pastoralists in the study area to trek animals long distances in search of pasture and water. In this situation, it was impractical to collect aborted fetuses and conduct post-mortem of dead animals that would complement our improved understanding of the vaccine and RVFV transmission dynamics in the area. It is important to note that the pregnant animals were included in the trial without characterizing the stage of pregnancy. Our inferences are made on vaccine safety without taking into account different stages of pregnancy. However, in our study, the vaccine-induced abortion remains unlikely, as there was no significant difference in the proportion of abortion between vaccinated and control animals. The quality of some of the clinical manifestations reported by animal owners/herders might have been affected by recall bias and/or preferences to report amongst them.

The serum samples were tested using only the ELISA kits. The results presented reflect the proportion of animals with antibodies and their persistence over time but not the antibody titres. Validation of the results using other tests such virus neutralization test is likely to improve understanding of antibody dynamics. We cannot account for the effect modification of natural infection on vaccine dynamics, which is important for estimation of the true efficacy of the vaccine. Considering the fact that the study was conducted in an area with a history of RVF outbreaks, our analysis is limited in that it cannot disentangle the effects of vaccine-induced immunity and naturally acquired protection, suggesting that development of a test to differentiate immunological response resulting from vaccination from that of natural infection is likely a breakthrough.

The assessment of the vaccine effectiveness against the clinical form of the disease over time is the most challenging aspect of the analysis. The clinical form of RVF was not observed during the period of the study, accordingly, making it difficult to directly account for the effectiveness of the vaccine against the clinical form of the disease. This study was however not designed to establish the causal relationship between vaccine as exposure and clinical RVF disease as the primary endpoint of interest. Therefore, the analysis outputs presented are meant to be descriptive rather than prescriptive, focusing on anti-RVFV antibody production dynamics as the primary endpoint. Further evaluation during periods of expected disease outbreaks with shorter sampling intervals than the one used in the study would enrich the current data. Besides the limitations, the study provides useful information on vaccine safety, antibodies production and their persistence, which could guide animal vaccination strategies.

## Conclusion

The findings of this study indicate that the vaccine was well-tolerated by sheep, goats, and cattle and there were no signs of local or general adverse effects. By the end of 360 days of follow-up, about 80.0% of animals from the vaccinated group mounted a serological response, with a peak IgG seropositivity detected 30 days post-inoculation. The vaccine is considered safe, with high immunogenicity in sheep and goats and moderate immunogenicity in cattle under field conditions. Further evaluation of the vaccine strategically before the expected RVF epidemic will provide additional data on the effectiveness of the vaccine.

## Data Availability Statement

The original contributions presented in the study are included in the article/supplementary material, further inquiries can be directed to the corresponding author.

## Ethics Statement

The animal study was reviewed and approved by Ministry of Agriculture, Livestock and Fisheries in Tanzania.

## Author Contributions

CS: conceptualization, supervision of clinical monitoring of animals, sample and data collection, data curation, formal analysis, and writing–original draft preparation. CS, EK, ES, LM, MR, JP, and JS: methodology, investigation, writing–critical review, editing, visualization, and interpretation of data. All authors approved the final version of the manuscript.

## Funding

This work is based on research funded in part by the Bill & Melinda Gates Foundation (Investment ID OPP-1009497) and with United Kingdom (UK) aid from the UK Government (GB-1-203188) through the Global Alliance for Livestock Veterinary Medicines. The findings and conclusions contained within are those of the authors and do not necessarily reflect positions or policies of the Bill and Melinda Gates Foundation or the UK Government.

## Conflict of Interest

The authors declare that the research was conducted in the absence of any commercial or financial relationships that could be construed as a potential conflict of interest.

## Publisher's Note

All claims expressed in this article are solely those of the authors and do not necessarily represent those of their affiliated organizations, or those of the publisher, the editors and the reviewers. Any product that may be evaluated in this article, or claim that may be made by its manufacturer, is not guaranteed or endorsed by the publisher.
